# Safety and Efficacy of Vedolizumab for the Patients With Gastrointestinal Acute Graft‐Versus‐Host Disease Refractory to Steroid Treatment After Allogeneic Hematopoietic Stem Cell Transplantation: A Real‐World, Retrospective Multicenter Study (VAST Study)

**DOI:** 10.1002/mco2.70942

**Published:** 2026-08-19

**Authors:** Leqing Cao, Yi Xia, Song Xue, Jianping Zhang, Jiameng Li, Yuewen Wang, Qi Wen, Ze Tian, Jun Kong, Daoxing Deng, Xiaohui Zhang, Yang Cao, Na Xu, Xiaodong Mo

**Affiliations:** ^1^ Peking University People's Hospital Peking University Institute of Hematology National Clinical Research Center for Hematologic Disease Beijing Key Laboratory of Cell and Gene Therapy for Hematologic Malignancies Peking University Beijing China; ^2^ Beijing Lu Daopei Hospital Beijing China; ^3^ Hebei Yanda Ludaopei Hospital Langfang Hebei Province China; ^4^ Peking University Health Science Center Peking University Beijing China; ^5^ Department of Hematology Tongji Hospital Tongji Medical College Huazhong University of Science and Technology Wuhan Hubei China; ^6^ Department of Hematology Nanfang Hospital Southern Medical University Guangzhou China

**Keywords:** acute graft‐versus‐host disease, allogeneic hematopoietic stem cell transplantation, gastrointestinal, steroid refractory, vedolizumab

## Abstract

Steroid‐refractory (SR) gastrointestinal (GI) acute graft‐versus‐host disease (aGVHD) is the major cause of early mortality after allogeneic hematopoietic stem cell transplantation. Vedolizumab could be used as the second‐ or third‐line treatment of GI‐aGVHD. Our real‐world, retrospective multicenter study, in which 100 SR‐GI‐aGVHD patients who received vedolizumab were enrolled, confirmed its effectiveness. Note that 411 SR‐GI‐aGVHD patients who received the best available treatments (BATs) during the same period were enrolled as controls. Inverse probability of treatment weighting (IPTW) and propensity score matching (PSM) were conducted as sensitivity analyses. The overall response rates (ORRs) for SR‐GI‐aGVHD on day 28 and at any time point were 68.0% and 81.0%, respectively, and the 2‐year probabilities of overall survival and nonrelapse mortality were 58.3% and 31.9%, respectively, after vedolizumab treatment. Multivariate analysis revealed that initiating vedolizumab treatment within 7 days after SR‐aGVHD onset could help improve the ORR. In the sensitivity analysis, compared with the BATs, vedolizumab improved the ORR and complete response rate, particularly in the stage 3–4 SR‐GI‐aGVHD subgroup, and it reduced the infection rate after IPTW and PSM adjustment. These findings confirmed the efficacy of vedolizumab in a real‐world setting, which can help to control SR‐GI‐aGVHD more effectively and safely.

## Graphical abstract

This multicenter, real‐world study with a large sample size (i.e., vedolizumab for aGVHD refractory to steroid treatment, VAST study) was designed to identify the safety and efficacy of vedolizumab treatment in patients with steroid‐refractory gastrointestinal acute graft‐versus‐host disease (SR‐GI‐aGVHD). The overall response rates (ORR) on day 28 and at any time point were 68.0% and 81.0%, respectively. In the sensitivity analysis, compared with the BATs, vedolizumab improved the ORR and complete response rate and reduced the infection rate. These findings confirmed the efficacy of vedolizumab in a real‐world setting, which can help to control SR‐GI‐aGVHD more effectively and safely.

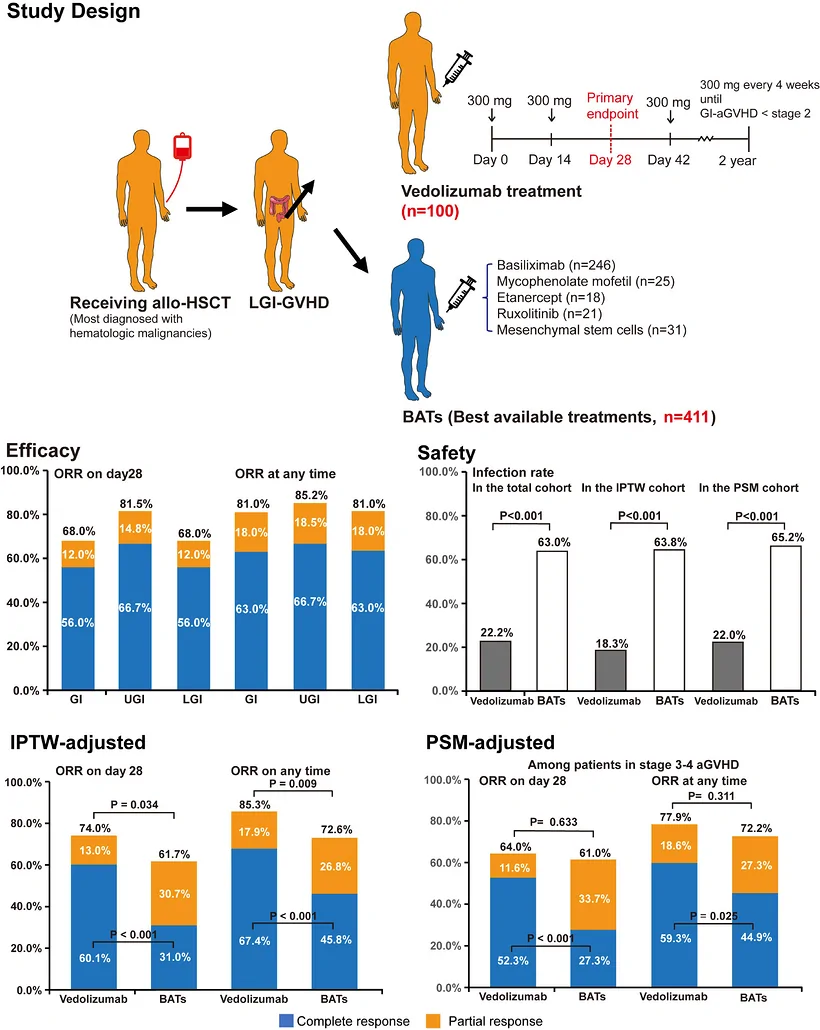

## 1 Introduction

Allogeneic hematopoietic stem cell transplantation (allo‐HSCT) is a curative modality for hematologic malignancies [1, 2, 3, 4]. The number of allo‐HSCTs in Europe exceeded 19,000 in 2022 [5], and the number in China has exceeded 15,000 since 2023 [6]. A human leukocyte antigen (HLA) identical sibling donor is the first choice for allo‐HSCT. With the development of transplant techniques, alternative donors, particularly haploidentical related donors (HIDs), have become important in China [6].

When immunocompetent T cells in the graft recognize the recipient (the host) as foreign, the donor T cells activate and attack the recipient to clear the foreign antigen‐bearing cells, which causes graft‐versus‐host disease (GVHD). Although several protocols, such as granulocyte colony‐stimulating factor (G‐CSF)‐ and ATG‐based regimens established by Peking University, as well as post‐transplant cyclophosphamide (PTCy) regimens established by Johns Hopkins University, have been widely used [7], severe acute GVHD (aGVHD) is still life threatening in the early stage of allo‐HSCT, particularly for those with gastrointestinal (GI) involvement [8]. GI‐aGVHD can cause water–electrolyte imbalance, malnutrition, GI hemorrhage, intestinal obstruction, and intestinal flora translocation [9], and the 2‐year nonrelapse mortality (NRM) rate can be as high as 62% for those with severe GI‐aGVHD [10].

Traditionally, corticosteroids are the first‐line treatment for aGVHD [11], and systemic immunosuppressive drugs (such as ruxolitinib and interleukin‐2 receptor [IL‐2R] antagonists) [12, 13, 14] are the foundation for the treatments of steroid refractory (SR)‐aGVHD. However, these treatments inevitably increase the risk of infection. Chen et al. [15] reported that the upregulation of α4β7 integrin expression on the surface of activated T cells was related to the subsequent occurrence of GI‐aGVHD. Moreover, several studies suggest that the trafficking of activated donor effector T cells to the gastrointestinal tract, where they induce tissue damage, is critical for the pathogenesis of GI‐aGVHD [16, 17]. Thus, the molecular interactions necessary for effector lymphocyte migration represent potential therapeutic targets for treating GI‐aGVHD [18, 19].

Vedolizumab is a monoclonal antibody against α4β7 integrin. By inhibiting the interaction between α4β7 and cell adhesion molecule‐1, vedolizumab can limit lymphocyte trafficking into gut‐associated lymphoid tissue and reduce intestinal inflammation [20], making it an important treatment for inflammatory bowel disease [21, 22]. Because lymphocyte homing is also involved in the development of GI‐aGVHD, vedolizumab has a biologic rationale for both prophylaxis [20, 23, 24] and treatment of GI‐aGVHD.

Several small studies have reported the efficacy of vedolizumab treatment for patients with SR‐GI‐aGVHD [25, 26, 27, 28, 29]. In a multicenter study with 29 SR‐GI‐aGVHD patients [30], the overall response rate (ORR) at 6–10 weeks was 64%, and overall survival (OS) at 6 months was 54% after vedolizumab treatment. In another study that included 29 patients with SR‐GI‐aGVHD, the ORR and complete response (CR) rate were 79% and 28%, respectively, after vedolizumab treatment. In the meta‐analysis conducted by Li et al. [31], the pooled ORRs at 14 days, 28 days, and 12 months were 60.5%, 50.0%, and 76.9%, respectively, and the pooled OS rates at 6 and 12 months were 34.5% and 29.2%, respectively, for GI‐aGVHD patients receiving vedolizumab treatment. However, some unresolved questions remain; for example, no studies have compared the efficacy and safety between therapeutic protocols with and without vedolizumab in SR‐GI‐aGVHD, and the influence of prior or concurrent second‐line therapies on the outcomes of vedolizumab treatment remains unclear. In addition, the predictors of response to vedolizumab should be further identified.

Thus, we conducted a multicenter, real‐world study with a large sample size (i.e., vedolizumab for aGVHD refractory to steroid treatment, VAST study) to comprehensively identify the safety and efficacy of vedolizumab treatment in patients with SR‐GI‐aGVHD.

## 2 Results

### 2.1 Patient Characteristics

In total, 100 patients who had SR‐LGI‐aGVHD and who received vedolizumab were included in this study (stage 2: 14.0%; stage 3: 51.0%; stage 4: 35.0%) (Table 1, Figure S1), and the characteristics of aGVHD are shown in Table S1. All 100 patients had LGI‐SR‐aGVHD, and 27 of them had upper GI (UGI)‐SR‐aGVHD simultaneously before vedolizumab treatment. The median interval from the diagnosis of SR‐aGVHD to the initiation of vedolizumab treatment was 13 days (range: 4–121 days). The median follow‐up time after vedolizumab treatment was 200 days (range: 20–1302 days).

**TABLE 1:** Patient characteristics.

	Before adjusted			IPTW adjusted			PSM adjusted		
Characteristic	Vedolizumab (*n* = 100)	BATs (*n* = 411)	*p*	Vedolizumab (*n* = 100)	BATs (*n* = 411)	*p*	Vedolizumab (*n* = 100)	BATs (*n* = 247)	*p*
Median age, year (range)	33 (2−66)	25 (1−63)	0.005	23 (2−66)	26 (1−63)	0.574	33 (2−66)	28 (1−63)	0.090
Female sex, *n* (%)	50 (50.0)	152 (37.0)	0.017	44.6	39.7	0.452	50 (50.0)	118 (47.8)	0.707
Disease at diagnosis, *n* (%)			0.124			0.183			0.097
Hematologic malignancies	91 (91.0)	346 (84.2)		90.5	84.5		91 (91.0)	208 (84.2)	
Nonmalignant hematologic disease	9 (9.0)	65 (15.8)		9.5	15.5		9 (9.0)	39 (15.8)	
Donor–recipient relationship, *n* (%)			0.203			0.946			0.353
MSD	73 (73.0)	283 (68.9)		68.8	68.1		73 (73.0)	173 (70.0)	
HID	16 (16.0)	62 (15.1)		16.4	16.0		16 (16.0)	44 (17.8)	
MUD	9 (9.0)	33 (8.0)		7.8	7.8		9 (9.0)	16 (6.5)	
Umbilical cord blood	2 (2.0)	33 (8.0)		7.0	8.0		2 (2.0)	14 (5.7)	
Donor‐recipient sex matched, *n* (%)			0.033			0.412			0.058
Others	90 (90.0)	333 (81.0)		92.5	88.7		90 (90.0)	202 (81.8)	
Female to male	10 (10.0)	78 (19.0)		7.5	11.3		10 (10.0)	45 (18.2)	
Conditioning regimen, *n* (%)			0.121			0.085			0.110
Chemotherapy‐based regimen	76 (76.0)	340 (82.7)		73.7	82.9		76 (76.0)	206 (83.4)	
TBI‐based regimen	24 (24.0)	71 (17.3)		26.3	17.1		24 (24.0)	41 (16.6)	
HCT‐CI score, *n* (%)			0.773			0.857			0.502
0	77 (77.0)	311 (75.7)		77.0	75.0		77 (77.0)	183 (74.1)	
1–2	20 (20.0)	81 (19.7)		19.5	20.1		20 (20.0)	49 (19.8)	
≥3	3 (3.0)	19 (4.6)		3.5	4.9		3 (3.0)	15 (6.1)	
Steroid refractory type, *n* (%)			0.761			0.878			1.000
Steroid resistant	88 (88.0)	357 (86.9)		87.6	87.0		88 (88.0)	219 (88.7)	
Steroid dependent	12 (12.0)	54 (13.1)		12.4	13.0		12 (12.0)	28 (11.3)	
Severity of GI‐aGVHD before treatment, *n* (%)			<0.001			0.979			0.386
Stage 2	14 (14.0)	154 (37.5)		33.2	32.9		14 (14.0)	42 (17.0)	
Stage 3	51 (51.0)	124 (30.2)		33.1	34.1		51 (51.0)	106 (42.9)	
Stage 4	35 (35.0)	133 (32.4)		33.8	33.0		35 (35.0)	99 (40.1)	

Abbreviations: BATs, best available treatments; GI‐aGVHD, gastrointestinal acute graft‐versus‐host disease; HCT‐CI, hematopoietic cell transplantation‐comorbidity index; HID, haploidentical donor; IPTW, inverse probability of treatment weighting; MSD, matched sibling donor; MUD, matched unrelated donor; PSM, propensity score matching; TBI, total body irradiation.

### 2.2 Response of Different Organs

#### 2.2.1 GI‐aGVHD

Among the 81 patients who achieved a response, the median time interval from vedolizumab treatment to response was 21 days (range: 3–122 days). The median number of doses of vedolizumab was one dose (range: 1–4), and 50 (61.7%), 26 (32.1%), three (3.7%), and two (2.5%) patients achieved a response after one dose, two doses, three doses, and four doses of vedolizumab, respectively. The ORRs on day 28 and at any time after vedolizumab treatment were 68.0% (95% CI, 57.9%–77.0%) and 81.0% (95% CI, 71.9%–88.2%), respectively, for patients with SR‐GI‐aGVHD.

**FIGURE 1:**
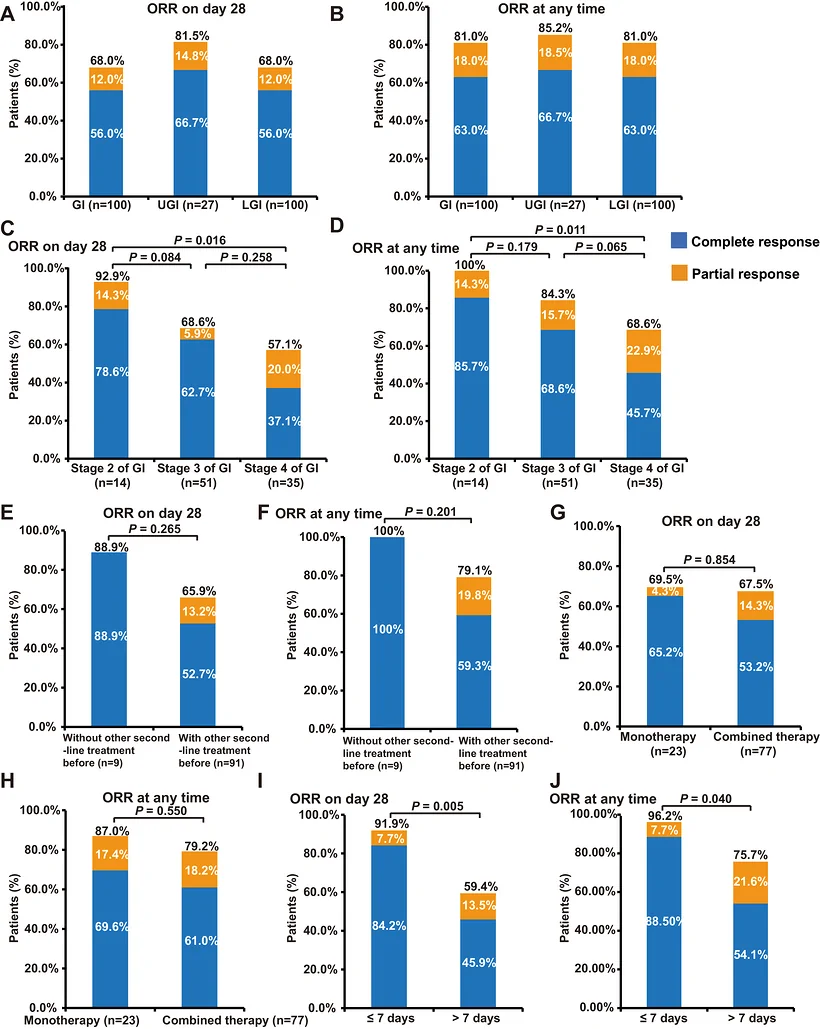
Overall response after vedolizumab treatment in patients with steroid‐refractory acute graft‐versus‐host disease (SR‐aGVHD). (A, B) Overall response of different involved organs on Day 28 (A) and at any time (B) after vedolizumab treatment. (C–J) Overall response on Day 28 and at any time in patients with steroid‐refractory gastrointestinal acute GVHD (SR‐GI‐aGVHD), stratified by GI‐aGVHD severity on Day 28 (C) and at any time (D), previous exposure to other second‐line treatments before vedolizumab on Day 28 (E) and at any time (F), vedolizumab monotherapy versus combined therapy on day 28 (G) and at any time (H), and the interval from SR‐aGVHD diagnosis to vedolizumab treatment initiation (≤ 7 days vs. > 7 days) on Day 28 (I) and at any time (J) after vedolizumab treatment. An overall response was defined as a complete response plus a partial response. Complete response and partial response are shown in blue and orange, respectively.

The ORRs on day 28 were 81.5% (95% CI, 57.7%–91.4%) and 68.0% (95% CI, 57.9–77.0%), respectively, for upper GI (UGI)‐ and LGI‐SR aGVHD, and the ORRs at any time point were 85.2% (95% CI 61.9%–93.7%) and 81.0% (95% CI 71.9%–88.2%), respectively, for UGI‐ and LGI‐SR aGVHD (Figure 1A,B). The ORRs on Day 28 after vedolizumab treatment were 92.9% (95% CI 66.1%–99.8%), 68.6% (95% CI 54.1%–80.9%), and 57.1% (95% CI 39.4%–73.7%), respectively, and the ORRs at any time point were 100% (95% CI 76.8%–100%), 84.3% (95% CI 71.4%–93.0%), and 68.6% (95% CI 50.7%–83.1%), respectively, for patients with stage 2, 3, and 4 SR‐GI‐aGVHD (Figure 1C,D).

The associations between the time interval from the diagnosis of SR‐aGVHD to vedolizumab treatment and the ORR are shown in Table S2, and the ORRs for stage 3–4 SR‐GI‐aGVHD patients who received vedolizumab treatment ≤ 5 days and > 5 or ≤7 days after the diagnosis of SR‐aGVHD were comparable. Baseline characteristics were comparable between the ≤ 7‐day and > 7‐day groups (Table S3).

Before starting vedolizumab, 91 patients had received at least one other second‐line therapy (Figure S2). In these patients, the ORRs on day 28 and at any time after vedolizumab treatment were 65.9% (95% CI, 55.3%–75.5%) and 79.1% (95% CI, 69.3%–86.9%), respectively, which were comparable to those without prior second‐line therapies (88.9% [95% CI, 51.8%–99.7%; *p* = 0.265] and 100.0% [95% CI, 66.4%–100.0%; *p* = 0.201], respectively) (Figure 1E,F). In particular, patients receiving ruxolitinib treatment before vedolizumab had Day 28 ORRs of 58.8% (95% CI, 44.2%–72.4%) and ORRs at any time after vedolizumab treatment of 80.4% (95% CI, 66.9%–90.2%) (Figure S2). Across all degrees of GI‐aGVHD severity, the corresponding ORRs remained comparable regardless of prior exposure to other second‐line therapies (Table S4).

A total of 23 patients received vedolizumab as monotherapy, and 77 patients received vedolizumab as an add‐on salvage therapy (i.e., combined therapy) (Figure S3). Stage 3–4 SR‐GI‐aGVHD was more frequent among patients receiving combined therapy (Table S5). Patients receiving combined therapy and those receiving monotherapy had comparable ORRs both on day 28 (67.5%, 95% CI, 55.9%–77.8% versus 69.5% 95% CI, 47.1%–86.8%; *p* = 0.854) and at any time (79.2%, 95% CI, 68.5%–87.6% versus 87.0%, 95% CI, 66.4%–97.2%; *p* = 0.550) after vedolizumab treatment (Figure 1G–H). Across all degrees of GI‐aGVHD severity, the corresponding ORRs were comparable between combined therapy and monotherapy groups (Table S4).

Multivariate analysis revealed that a time interval > 7 days from SR‐aGVHD diagnosis to vedolizumab treatment (*p =* 0.005) and stage 3–4 SR‐GI‐aGVHD before vedolizumab treatment (*p =* 0.042) increased the risk of nonresponse on Day 28, whereas only the time interval from SR‐aGVHD diagnosis to vedolizumab treatment increased the risk of nonresponse at any time (Table 2, Table S6, Figure 1I–J).

**TABLE 2:** Multivariate analysis of response rates for gastrointestinal aGVHD and overall aGVHD after vedolizumab treatment.

	HR (95% CI)	*p*
**GI‐aGVHD**		
Lack of response on Day 28		
Time from SR‐aGVHD to vedolizumab treatment		
> 7 days	1.00	
≤ 7 days	0.11 (0.02−0.51)	0.005
Severity of GI‐aGVHD before vedolizumab treatment		
Stage 2	1.00	
Stage 3–4	8.85 (1.08−72.67)	0.042
Lack of response at any time		
Time to vedolizumab after aGVHD		
> 7 days	1.00	
≤ 7 days	0.11 (0.01−0.91)	0.040
**Overall aGVHD**		
Lack of response on Day 28		
Severity of aGVHD before vedolizumab treatment		
Grade II	1.00	
Grade III to IV	8.85 (1.08−72.67)	0.042
Time from SR‐aGVHD to vedolizumab treatment		
> 7 days	1.00	
≤ 7 days	0.11 (0.02−0.51)	0.005

*Note*: No factors for overall aGVHD response at any time were identified.

Abbreviation: GI‐aGVHD, gastrointestinal acute graft‐versus‐host disease.

#### 2.2.2 Skin and Liver SR‐aGVHD

We explored the effectiveness of vedolizumab treatment in non‐GI organs. Twenty‐eight and 12 patients had SR‐skin GVHD and SR‐liver GVHD, respectively, before vedolizumab treatment. The ORRs after vedolizumab treatment on Day 28 were 71.4% (95% CI 52.9%−84.4%) and 50.0% (95% CI 25.4%−74.6%), respectively, while the ORRs at any time point were 85.7% (95% CI 68.5%−94.3%) and 66.7% (95% CI 35.4%−88.7%), respectively, for patients with SR‐skin‐ and SR‐liver‐aGVHD (Figure S4).

### 2.3 Overall aGVHD Response

The ORR, CR rate, and PR rate on Day 28 after vedolizumab treatment were 68.0% (95% CI 58.9%–77.1%), 56.0% (95% CI 46.3%–65.7%), and 12.0% (95% CI 5.6%–18.4%), respectively. The ORR, CR, and PR at any time after vedolizumab treatment were 81.0% (95% CI 73.3%–88.7%), 63.0% (95% CI 53.5%–72.5%), and 18.0% (95% CI 10.5%–25.5%), respectively. Compared with patients with grade III to IV aGVHD, patients with grade II aGVHD before vedolizumab treatment had a higher ORR on Day 28 (92.9%, 95% CI, 66.1–99.8% versus 63.9%, 95% CI, 52.9%–74.0%; *p =* 0.042). Moreover, compared with patients with grade III to IV aGVHD, patients with grade II aGVHD tended to have a higher ORR at any time (100.0%, 95% CI, 76.8%–100.0% versus 77.9%, 95% CI, 67.7%–86.1%; *p =* 0.051). High‐ or standard‐risk refined Minnesota aGVHD risk scores before vedolizumab treatment were not correlated with the ORRs on Day 28 and at any time (Figure S5).

Multivariate analysis revealed that the time interval from SR‐aGVHD diagnosis to vedolizumab treatment (≤ 7 days vs. >7 days, *p =* 0.005) and grade III to IV aGVHD before vedolizumab treatment (*p =* 0.042) were associated with the ORR on Day 28, but no variables contributed to the lack of overall response at any time (Table 2, Table S6).

### 2.4 CGVHD After Vedolizumab Treatment

By the end of follow‐up, 22 patients had developed cGVHD (Table S7), and the median time from vedolizumab initiation to cGVHD onset was 77 (range 26–473) days. The 1‐year cumulative incidence rates of total and moderate‐to‐severe cGVHD after vedolizumab treatment were 29.6% (95% CI 17.4%–41.8%) and 17.9% (95% CI 8.5%–27.3%), respectively.

### 2.5 Toxicities and New‐Onset Infections Following Vedolizumab Initiation

No infusion‐related or allergic reaction was observed. The rates of new‐onset bacterial, viral, and fungal infections were 4.0% (95% CI 1.3%–10.5%), 19.0% (95% CI 12.1%–28.3%), and 4.0% (95% CI 1.3%–10.5%), respectively, after vedolizumab treatment (Table 3). Overall, 22.0% of patients (95% CI, 14.6%–31.6%) developed at least one type of infection, and 3.0% (95% CI, 1.0%–8.5%) developed two or more types. The corresponding rates in patients with stage 2 versus stage 3–4 GI‐aGVHD were 0% (95% CI, 0–23.2%) versus 25.6% (95% CI, 16.8–36.1%; *p* = 0.032) and 0% (95% CI, 0–23.2%) versus 3.5% (95% CI, 1.2–9.8%; *p* = 1.000), respectively (Table S8). Patients receiving vedolizumab as an add‐on salvage therapy had a higher rate of ≥ 1 infection type than those receiving vedolizumab monotherapy (27.3%, 95% CI, 17.7–38.6% versus 4.3%, 95% CI, 0.8–21.0%*; p* = 0.020) (Table S8).

**TABLE 3:** Infections documented following vedolizumab initiation.

Infection type	*n* (%)
Bacterial infection	4 (4.0)
Sepsis	1 (1.0)
Pneumonia	3 (3.0)
Viral infection	19 (19.0)
Cytomegalovirus DNAemia	15 (15.0)
Epstein‐Barr virus DNAemia	12 (12.0)
Fungal infection	4 (4.0)
Pneumonia	4 (4.0)
At least one infection type (≥ 1 type)	22 (22.0)
Two or more infection types (≥ 2 types)	3 (3.0)

Multivariate analysis revealed that liver involvement at aGVHD diagnosis (HR, 10.46; 95% CI 2.10–52.14; *p =* 0.004) and receiving vedolizumab as an add‐on salvage therapy (HR, 1.18, 95% CI, 1.02–1.36; *p =* 0.023) were correlated with a higher risk of any infection (≥ 1 event). No factor was correlated with the risk of ≥ 2 types of infection (Table 4, Table S6).

**TABLE 4:** Multivariate analysis of clinical outcomes after vedolizumab treatment.

	HR (95% CI)	*p*
**Any infection (≥ 1 type)**		
Liver involvement at aGVHD diagnosis		
No	1.00	
Yes	10.46 (2.10−52.14)	0.004
Type of vedolizumab treatment		
Monotherapy	1.00	
Combined therapy	1.18 (1.02−1.36)	0.023
**Treatment failure as defined by OS**		
Liver involvement before vedolizumab treatment		
No	1.00	
Yes	4.24 (2.10−8.56)	< 0.001
**Treatment failure as defined by DFS**		
Liver involvement before vedolizumab treatment		
No	1.00	
Yes	3.99 (1.99−8.00)	< 0.001
**Non‐relapse mortality**		
Liver involvement before vedolizumab treatment		
No	1.00	
Yes	4.12 (1.82−9.29)	0.001

*Note*: No factors for ≥ 2 types of infection or relapse were identified.

Abbreviations: aGVHD, acute graft‐versus‐host disease; DFS, disease‐free survival. OS, overall survival.

### 2.6 Other Clinical Outcomes

The 2‐year probabilities of OS and DFS were 58.3% (95% CI, 46.5–70.1%) and 51.9% (95% CI, 37.8–66.0%), whereas the 2‐year cumulative incidences of relapse and NRM were 16.2% (95% CI, 4.2–28.2%) and 31.9% (95% CI, 21.1–42.7%), respectively. The causes of mortality are summarized in Table S9. In the multivariate analysis, only liver involvement before vedolizumab treatment was associated with OS, DFS, and NRM. In particular, high‐risk Minnesota disease and grade III to IV aGVHD were not associated with NRM or survival (Table 4, Table S6).

### 2.7 Comparison Between Vedolizumab and BATs in the Historical Cohort

#### 2.7.1 Response After Treatment

We compared vedolizumab with the BATs for patients with SR‐GI‐aGVHD during the same period (Table 1). The vedolizumab group presented comparable ORRs on Day 28 and at any time point compared with BATs group. In patients who had stage 3–4 GI‐aGVHD, although the corresponding ORRs were comparable, the vedolizumab group presented a significantly higher CRR on Day 28 (52.3%, 41.3%–63.2% vs. 24.9%, 19.7%–30.7%; *p* < 0.001) and at any time (59.3%, 95% CI, 48.2%–69.8% vs. 41.2%, 95% CI, 35.2%–47.5%, *p =* 0.004) than the BATs group (Figure S6). Table S10 summarizes the comparisons of clinical outcomes between vedolizumab and each intervention included in the BATs group.

**FIGURE 2:**
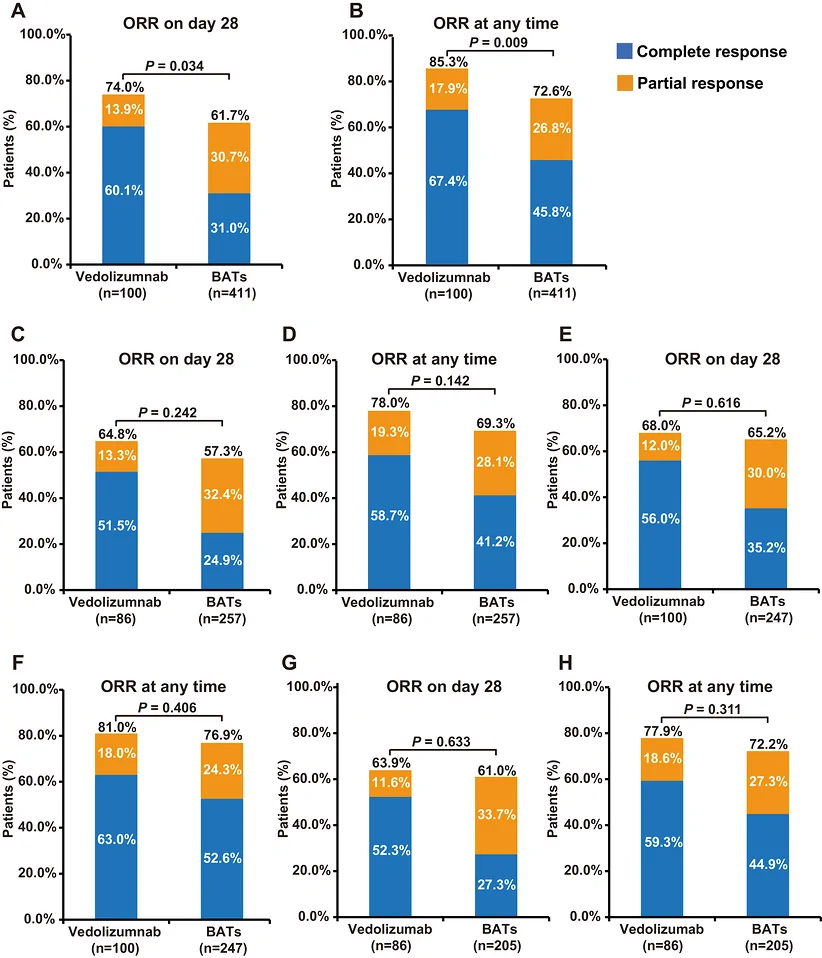
Overall response after vedolizumab versus best available treatments (BATs) in patients with steroid‐refractory gastrointestinal acute graft‐versus‐host disease (SR‐GI‐aGVHD) after adjustment. (A, B) Overall response rate (ORR) on Day 28 (A) and at any time (B) after vedolizumab versus BATs in the total cohort after inverse probability of treatment weighting (IPTW) analysis. (C, D) ORR on Day 28 (C) and at any time (D) after vedolizumab versus BATs in patients with stage 3–4 gastrointestinal GVHD after IPTW analysis. (E, F) ORR on Day 28 (E) and at any time (F) after vedolizumab versus BATs in the total cohort after propensity score matching (PSM) analysis. (G, H) ORR on Day 28 (G) and at any time (H) after vedolizumab versus BATs in patients with stage 3–4 gastrointestinal GVHD after PSM analysis. An overall response was defined as a complete response plus a partial response. Complete response and partial response are shown in blue and orange, respectively.

After inverse probability of treatment weighting (IPTW) and propensity score matching (PSM) adjustment, the characteristic parameters were well distributed in the two groups (Table 1). In the sensitivity analysis using IPTW analysis, the vedolizumab group presented a higher ORR (on Day 28: 74.0%, 95% CI, 63.5%–82.3% vs. 61.7%, 95% CI, 56.8%–66.4%, *p =* 0.034; at any time, 85.3%, 95% CI, 76.9%–91.0% vs. 72.6%, 95% CI, 68.0%–76.7%, *p =* 0.009) and a higher CRR (on Day 28: 60.1%, 95% CI, 48.2%–70.9% vs. 31.0%, 95% CI, 26.7%–35.7%; *p* < 0.001; at any time, 67.4%, 95% CI, 56.0%–77.1% vs. 45.8%, 95% CI, 41.0–50.7%; *p* < 0.001) than those in the BATs group. In patients with stage 3–4 GI‐aGVHD, the vedolizumab group presented a better CRR on Day 28 (51.5%, 95% CI, 40.1%–62.7% vs. 24.9%, 95% CI, 19.9%–30.6%; *p* < 0.001) and at any time (58.7%, 95% CI, 47.2%–69.4% vs. 41.2%, 95% CI, 35.3%–47.4%; *p =* 0.008) than the BATs group (Figure 2).

In the sensitivity analysis using PSM analysis, the ORRs on Day 28 and at any time were comparable between the vedolizumab and BATs groups. However, the vedolizumab group presented a higher CRR (on Day 28: 56.0%, 95% CI, 45.7%–65.9% vs. 35.2%, 95% CI, 29.3%–41.5%; *p* < 0.001; at any time: 63.0%, 95% CI, 52.8%–72.4% vs. 52.6%, 95% CI, 46.2%–59.0%; *p =* 0.078) than that in the BATs group. In patients with stage 3–4 GI‐aGVHD, the vedolizumab group presented a better CRR on Day 28 (52.3%, 95% CI, 41.3%–63.2% vs. 27.3%, 95% CI, 21.3%–34.0%; *p* < 0.001) and at any time (59.3%, 95% CI, 48.2%–69.8% vs. 44.9%, 95% CI, 37.9%–52.0%; *p =* 0.025) compared with the BATs group (Figure 2).

#### 2.7.2 Infections After Treatment

The rate of occurring ≥ 1 type of infection was significantly lower in vedolizumab group than that in the BATs group (22.2%, 95% CI: 14.3%–31.4% vs. 63.0%, 95% CI: 58.1%–67.7%; *p* < 0.001). In the sensitivity analyses, the corresponding rate remained significantly lower in the vedolizumab group after IPTW adjustment (18.3%, 95% CI: 11.6%–27.7% vs. 63.8%, 95% CI: 59.0%–68.4%; *p* < 0.001) and PSM (22.0%, 95% CI: 14.3%–31.4% vs. 65.2%, 95% CI: 58.9%–71.1%; *p* < 0.001) (Figure S7).

#### 2.7.3 Relapse, NRM, and Survival After Treatment

The 2‐year OS and DFS probabilities and the cumulative incidences of relapse and NRM were comparable between the vedolizumab and BATs groups (Figure S8) and were also similar in the IPTW analysis (Figure S9) and in the PSM analysis (Figure S10).

## 3 Discussion

In this large‐scale multicenter study, the ORRs on Day 28 and at any time were 68.0% and 81.0%, respectively, for patients with SR‐GI‐aGVHD. In addition, compared with control patients, patients who received vedolizumab therapy had a better ORR and a better CRR after IPTW adjustment, a better CRR after PSM adjustment, and a lower infection rate in both the IPTW and the PSM analysis. Thus far, this is the largest comprehensive study confirming that vedolizumab treatment is effective and safe for patients with SR‐GI‐aGVHD in a real‐world setting.

SR‐aGVHD is a common complication after allo‐HSCT; however, it remains an orphan disease with a limited number of patients, preventing the need for larger clinical studies [32, 33]. Although randomized controlled trials (RCTs) provide the most rigorous clinical evidence, the clinical situations of SR‐GI‐aGVHD patients are often complicated [8], and highly selective enrollment may limit the applicability of trial findings to routine practice. On the contrary, real‐world studies are closely related to clinical practice and are important for advancing therapies for SR‐GI‐aGVHD.

In the present study, the ORRs after vedolizumab treatment on Day 28 and at any time were 81.5% and 85.2%, respectively, for UGI‐aGVHD and were 68.0% and 81.0%, respectively, for LGI‐aGVHD. In the study of Fløisand et al. [30], the ORR at 6–10 weeks after vedolizumab initiation was 64% for SR‐GI‐aGVHD, and the ORR at 28 days was 50% in the meta‐analysis of Li et al. [31]. One explanation for the better response in our present study is that we used vedolizumab earlier after the diagnosis of SR‐aGVHD. The median duration from SR‐aGVHD diagnosis to the initiation of vedolizumab treatment was 13 days, which seemed to be earlier than the 21 days reported in the study of Fløisand et al. [30]. The GI tract plays a key role in this cytokine storm, as well as in the amplification of chemokines and the production of danger signals such as pathogen‐associated molecular patterns [34]. Blocking inflammation in the GI tract as early as possible may help to control SR‐GI‐aGVHD more easily. In addition, we observed that receiving other second‐line treatments before vedolizumab treatment increased the risk of infection, which also suggested that early use of vedolizumab might be important in patients with SR‐GI‐aGVHD.

Stage 3–4 GI‐aGVHD is a prognostic factor for a lower ORR and poorer survival [35, 36]. In the present study, although stage 3–4 SR‐GI‐aGVHD was associated with a lower ORR on Day 28, it did not affect the ORR at any time, NRM, or survival. These findings suggested that vedolizumab could partially overcome the poor prognosis of severe GI‐aGVHD.

As mentioned above, early use of vedolizumab could help to improve the efficacy of treatment for SR‐GI‐aGVHD. Similarly, a randomized phase 3 trial reported that vedolizumab could prevent GI‐aGVHD after allo‐HSCT [23], which was further confirmed by a Japanese subgroup analysis [24]. Thus, we suggest that combining vedolizumab with steroids as a first‐line therapy may be reasonable for patients with severe GI‐aGVHD, which should be further confirmed in the future.

According to the results of the REACH2 study [12] and several meta‐analyses [37, 38, 39], ruxolitinib has been recommended as a preferred treatment for SR‐aGVHD [11]. However, for those who failed to ruxolitinib treatment, optimal salvage treatment was unclear. In the present study, the ORRs on day 28 and at any time after vedolizumab treatment were 58.8% and 80.4%, respectively, for those who failed to ruxolitinib treatment. Thus, vedolizumab might be used as a potential salvage treatment for ruxolitinib in SR‐GI‐aGVHD.

However, the sample of patients who received ruxolitinib for the treatment of SR‐aGVHD was relatively small (*n =* 21) in the BATs group. This is because many physicians are worried that severe diarrhea may reduce the absorption of oral ruxolitinib in the GI tract and negatively impact its effectiveness. Thus, they were prone to use immunosuppressive drugs intravenously for LGI‐aGVHD patients. In addition, ruxolitinib was approved for the treatment of SR‐aGVHD by the China National Medical Products Administration (NMPA) in 2023. Finally, the multicenter phase 3 trial (REACH2 study) did not enroll Chinese patients with SR‐aGVHD, and a postmarketing study is currently underway (NCT06462469) in China. Thus far, the Chinese expert consensus does not recommend ruxolitinib as a preferred treatment for SR‐aGVHD [40] because no high‐level clinical evidence has confirmed its efficacy in China. Therefore, conclusions regarding the superiority of vedolizumab should be interpreted with caution and limited to the context of a heterogeneous BATs population.

For SR‐GI‐aGVHD patients who showed an unsatisfactory response to vedolizumab treatment, several methods may be used as salvage treatments. For example, in the RELAX study, the ORRs on Day 28 and at any time after xenopax treatment (a humanized IL‑2R antagonist) were 69.4% and 89.8%, respectively, for SR‐aGVHD patients who showed an unsatisfactory response to ruxolitinib‐based treatment [41]. In addition, the use of an IL‐2 receptor monoclonal antibody combined with MSCs might be a useful method for treating patients with SR‐aGVHD [42, 43], particularly for those with intestinal involvement [44, 45].

In addition to GI involvement, skin and liver aGVHD could be alleviated after vedolizumab treatment. Similarly, Waldman et al. [46] reported that in murine HSCT models, targeting β7 integrin was associated with a decrease in donor T‐cell infiltration of the liver and an overall significant decrease in hepatic GVHD. In addition, some patients received vedolizumab with prior second‐line treatments. The ability of vedolizumab to control GI‐aGVHD could weaken the cytokine storm, help achieve immune system homeostasis, and facilitate the control of aGVHD by other immunosuppressants.

Infection was the most important complication after second‐line treatment for aGVHD. In the REACH2 study, the rates of at least 1 infection were 79.6% and 69.3%, respectively, for ruxolitinib and BATs (such as mycophenolate mofetil, antithymocyte globulin, and infliximab). Unlike the traditional systemic immunosuppressants, vedolizumab disrupts the homing of alloreactive T cells and transmigration to GI lymphoid tissue without causing severe systemic immunosuppression. Therefore, the safety of the vedolizumab‐based protocol is more satisfactory than that of traditional immunosuppressive agents. In the present study, the rate of at least one infection event in the vedolizumab group was only 22.0%, which was significantly lower than that in the BATs group, suggesting that these patients might not need to adopt a more intensive anti‐infection prophylaxis strategy.

This study has some limitations. First, more than 70% of patients received vedolizumab as an add‐on salvage treatment for those who did not respond to prior second‐line therapies. Thus, we could not completely rule out the influence of other treatments for aGVHD. However, we observed that vedolizumab changed the process of SR‐GI‐aGVHD and that patients who received vedolizumab‐based therapy had a better response rate than those who did not receive vedolizumab according to the sensitivity analysis. Second, the BATs group was heterogeneous and included different therapies. This reflected the complexity of the SR‐GI‐aGVHD treatment in the real‐world setting; however, in the other RCTs for SR‐GVHD [12, 47], BATs including different second‐line therapies were also used as controls. Finally, despite IPTW/PSM, residual confounding is inevitable, such as the disease trajectory before vedolizumab, timing and sequence of prior therapies, and center‐specific treatment practices, which should be further identified in prospective RCTs.

In summary, this real‐world analysis confirmed the efficacy of vedolizumab treatment, which could help to control SR‐GI‐aGVHD more effectively and safely. In future prospective RCTs, further comparisons of the clinical outcomes between therapies with and without vedolizumab should be performed in SR‐GI‐aGVHD patients, which could further support our results.

## 4 Methods

### 4.1 Study Design

This was a retrospective multicenter cohort study conducted at five hospitals in China (see the participating units and investigators) and enrolled patients with GI‐aGVHD receiving vedolizumab treatment between March 1, 2019, and November 30, 2024. The final follow‐up was conducted on June 30, 2025; patients without complete follow‐up were censored at the date of their last available assessment.

### 4.2 Subjects and Procedures

Each center screened post‐allo‐HSCT recipients who had received at least one dose of vedolizumab after allo‐HSCT. The inclusion criteria for the final analysis were as follows: (1) diagnosed with grade II to IV aGVHD, with stage 1 to 4 GI involvement; and (2) patients were steroid resistant or steroid dependent according to international recommendations [48]. The exclusion criteria were as follows: (1) receiving vedolizumab treatment for GVHD prophylaxis, (2) receiving vedolizumab treatment as a first‐line treatment for aGVHD, and (3) having overlap syndrome.

**FIGURE 3:**
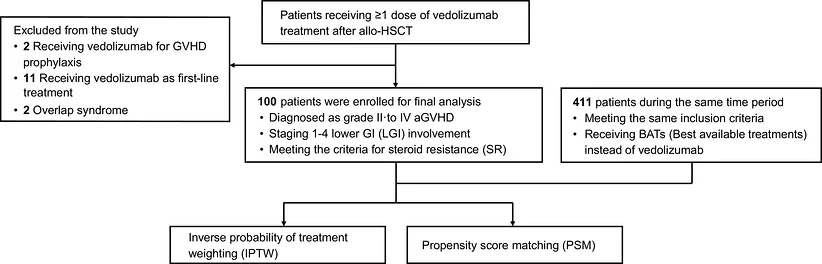
Flow diagram of the study. aGVHD, acute graft‐versus‐host disease; Allo‐HSCT, allogeneic hematopoietic stem cell transplantation; LGI, lower gastrointestinal.

This study also enrolled SR‐GI‐aGVHD patients who received best available treatments (BATs, *n =* 411) without vedolizumab during the same time period as the control. These patients should meet the same inclusion and exclusion criteria as the vedolizumab group. In the BATs group, 341 patients received monotherapies, including basiliximab (*n =* 246), mycophenolate mofetil (*n =* 25), etanercept (*n =* 18), ruxolitinib (*n =* 21), and mesenchymal stem cells (MSCs, *n =* 31). In addition, 64 and 6 patients received two‐ and three‐drug therapies, respectively (Figure 3).

### 4.3 Data Collection

Required information was obtained through institutional electronic medical records (Supporting Information Methods), which was independently reviewed by two physicians with extensive experience in allo‐HSCT.

Transplantation regimens were performed mainly according to recommendations from the Chinese Society of Hematology [40]. Particularly, GVHD prophylaxis consisted of a calcineurin inhibitor, mycophenolate mofetil, and short‐course methotrexate. Antithymocyte globulin was used for recipients of haploidentical or unrelated‐donor HSCT.

Vedolizumab (Entyvio; Takeda Pharmaceutical Co., Ltd., Tokyo, Japan) was administered at an initial dose of 300 mg i.v. at week 0, followed by the same dose at weeks 2 and 6. If the patients achieved a partial response (PR) within 6 weeks, vedolizumab treatment could be repeated every 4 weeks until the patient's GI‐aGVHD was less than stage 2. The efficacy of vedolizumab treatment was evaluated in accordance with international guidelines [49]. The selection of BATs strategies was guided by the Chinese expert consensus [40]. Steroid tapering was determined according to each center's experience. Vedolizumab monotherapy was defined as using vedolizumab alone at diagnosis of SR‐aGVHD, or as the sole salvage therapy after prior second‐line treatments. Combined therapy was defined as using vedolizumab as an add‐on salvage treatment after other second‐line treatments. Use of nonabsorbable oral steroids was not considered combined therapy.

### 4.4 Definition

The primary endpoint was the ORR on Day 28 for lower GI (LGI)‐aGVHD, defined as the proportion of patients achieving a CR or partial response (PR) (Supporting Information Methods). Response was assessed at least weekly after the first vedolizumab dose, according to the maximum aGVHD stage and grade in each involved organ and was required to persist for at least 3 weeks without additional systemic therapy. Secondary endpoints included organ‐specific response, ORR of overall aGVHD, incidence rate of cGVHD [50], OS, DFS, NRM, and relapse. Safety evaluation focused on adverse events, particularly infectious complications.

### 4.5 Statistical Analysis

The target sample size was calculated using the previously reported 28‐day ORR for LGI‐SR‐aGVHD by Biavasco et al. [51]. The study was designed to detect a 15% absolute increase in day‐28 ORR, corresponding to an expected ORR of 74.8% versus a reference rate of 59.8%, with type I and type II error rates set at 5% and 15%, respectively. After allowing for a 10% expulsion rate, 100 patients were planned for inclusion.

Continuous variables were compared with Student's *t* test, and categorical variables were analyzed using the *χ* ^2^ or Fisher's exact test. The time from SR‐aGVHD diagnosis to vedolizumab treatment was categorized as ≤ 7 days versus > 7 days and exploratory analyses were performed to compare earlier cutoff values. Survival was estimated by the Kaplan–Meier method, whereas ORR, cGVHD, NRM, and relapse were analyzed using competing‐risk methods [52]. Cox proportional hazards models were used to estimate HRs for ORR and other clinical outcomes. Variables with *p* > 0.1 were removed stepwise, and *p* < 0.05 was considered statistically significant (Supporting Information Methods).

Sensitivity analyses were performed using IPTW and PSM to adjust for age, sex, and severity of LGI‐aGVHD before second‐line treatment. For IPTW, stabilized IPTW weights were derived from the propensity score and reflected the probability of receiving the treatment actually administered [53]. Weighted response rates were estimated, and between‐group differences after IPTW were evaluated using logistic regression. For PSM, nearest neighbor method paired vedolizumab and BAT recipients within a caliper of 0.02. Analyses were conducted with Power Analysis and Sample Size software (PASS 23.0.4) and R software (v3.6.2, http://www.r‐project.org).

## Author Contributions

L.C. and Y.X. were responsible for writing the original draft, data analysis, and figure preparation. S.X. and J.Z. contributed to study design and critically revised the manuscript for important intellectual content. J.L. was responsible for data curation. Y.W., Q.W., Z.T., J.K., D.D., and X.Z. contributed to investigation, interpreted the results, and provided feedback on the manuscript. Y.C. and N.X. contributed to study design and critically revised the manuscript. X.M. was responsible for conceptualization, study design, funding acquisition, supervision, and critical revision of the manuscript. All authors reviewed and approved the final manuscript.

## Funding

This work was supported by the Natural Science Foundation of Beijing (Z230016), the National Natural Science Foundation of China (82570262), and the Fundamental Research Funds for Central Universities.

## Ethics Statement

The study was approved by the Institutional Review Board of Peking University People's Hospital (2026PHB248‐001) and conducted in accordance with the Declaration of Helsinki.

## Conflicts of Interest

The authors declare no conflicts of interest.

## Supporting information




**Supporting File 1**: mco270942‐sup‐0001‐SuppMat.docx

## Data Availability

The data of this study are available on reasonable request from the corresponding author.
