# Supplementary material for: Safety and Efficacy of Vedolizumab for the Patients With Gastrointestinal Acute Graft‐Versus‐Host Disease Refractory to Steroid Treatment After Allogeneic Hematopoietic Stem Cell Transplantation: A Real‐World, Retrospective Multicenter Study (VAST Study)
Source: MedComm (2020). 2026 Aug 19;7(9):e70942. doi: 10.1002/mco2.70942 (PMC13490922; doi:10.1002/mco2.70942)
Supplement: Supplementary file 1 [file MCO2-7-e70942-s001.docx]

**Title page**

**Title:** Safety and Efficacy of Vedolizumab for the Patients with Gastrointestinal Acute Graft-versus-host Disease Refractory to Steroid Treatment after Allogeneic Hematopoietic Stem Cell Transplantation: A Real-world, Retrospective Multicenter Study (**VAST** study)

**Short running title:** Vedolizumab for SR-GI-aGVHD

**Authors:** Leqing Cao^1#^, Yi Xia^1#^, Song Xue^2#^, Jianping Zhang^3#^, Jiameng Li^4#^, Yuewen Wang^1^, Qi Wen^1^, Ze Tian^4^, Jun Kong^1^, Daoxing Deng^1^, Xiaohui Zhang^1^, Yang Cao^5*^, Na Xu^6*^, Xiaodong Mo^1*^

**Authors' institutions:**

^1^Peking University People’s Hospital, Peking University Institute of Hematology, National Clinical Research Center for Hematologic Disease, Beijing Key Laboratory of Cell and Gene Therapy for Hematologic Malignancies, Peking University, Beijing 100044, China.

^2^Beijing Lu DaoPei Hospital, Beijing 101102, China.

^3^Hebei Yanda Ludaopei hospital, Langfang, Hebei Province 056200, China.

^4^Peking University Health Science Center, Peking University, Beijing 100191, China.

^5^Department of Hematology, Tongji Hospital, Tongji Medical College, Huazhong University of Science and Technology, Wuhan, Hubei 430030, China.

^6^Department of Hematology, Nanfang Hospital, Southern Medical University, Guangzhou 510000, China.

^#^Leqing Cao, Yi Xia, Song Xue, Jianping Zhang, and Jiameng Li contributed equally to this work.

**^*^Corresponding author:**

Yang Cao,

Department of Hematology, Tongji Hospital, Tongji Medical College, Huazhong University of Science and Technology, Wuhan, Hubei 430030, China. E-mail: [caoyangemma@163.com](mailto:caoyangemma@163.com)

Na Xu,

Department of Hematology, Nanfang Hospital, Southern Medical University, Guangzhou 510000, China. E-mail: [sprenaa@163.com](mailto:sprenaa@163.com);

Xiaodong Mo,

Peking University People’s Hospital, Peking University Institute of Hematology, National Clinical Research Center for Hematologic Disease, Beijing Key Laboratory of Cell and Gene Therapy for Hematologic Malignancies, Peking University, Beijing 100044, China. E-mail: moxiaodong@pkuph.edu.cn

**Vedolizumab for steroid-refractory gastrointestinal acute graft-versus-host disease: A real-world VAST study**

**Supplemental Material**

**Contents**

[Supplemental Methods	4](#_Toc234506101)

[Supplemental Tables	5](#_Toc234506102)

[Table S1. Characteristics of GVHD of patients receiving vedolizumab.	5](#_Toc234506103)

[Table S2. The association between time interval from SR-aGVHD diagnosis to vedolizumab treatment and ORR	7](#_Toc234506104)

[Table S3. Patient characteristics between those receiving vedolizumab treatment ≤ 7 days and > 7 days after SR-aGVHD diagnosis	8](#_Toc234506105)

[Table S4. The overall response on day 28 and at any time of different subgroups in patients with gastrointestinal (GI) involvement.	9](#_Toc234506106)

[Table S5. Patient characteristics between monotherapy and combined therapy groups.	10](#_Toc234506107)

[Table S6. Univariate analysis for response of GI-aGVHD and clinical outcomes after vedolizumab treatment.	11](#_Toc234506108)

[Table S7. Characteristics of cGVHD.	16](#_Toc234506109)

[Table S8. New onset infections after vedolizumab treatment in subgroup analysis.	17](#_Toc234506110)

[Table S9. Causes of death.	19](#_Toc234506111)

[Table S10. Comparison of overall response rate between vedolizumab and each individual intervention in the BATs group.	20](#_Toc234506112)

[Supplemental Figures	21](#_Toc234506113)

[Figure S1. Swimmers plot showing duration of treatment response (complete response or partial response) and survival status in patients treated with vedolizumab. Each horizontal bar represents one patient; bar length indicates time from vedolizumab initiation to last follow-up or event.	21](#_Toc234506114)

[Figure S2. Overall response rate (ORR) of patients receiving other second-line treatments before vedolizumab on day 28 (A) and at any time (B). Overall response included complete response (blue) plus partial response (orange) rates.	22](#_Toc234506115)

[Figure S3. Overall response rate (ORR) of patients who received other second-line treatments combined with vedolizumab on day 28 (A) and at any time (B). ORR included complete response (blue) plus partial response (orange) rates	23](#_Toc234506116)

[Figure S4. Overall response rate (ORR) of skin and liver after vedolizumab on day 28 (A) and at any time (B). Overall response included complete response (blue) plus partial response (orange) rates.	24](#_Toc234506117)

[Figure S5. Overall response rate (ORR) of patients with different severity of overall aGVHD after vedolizumab on day 28 and at any time. ORR included complete response (blue) plus partial response (orange) rates.	25](#_Toc234506118)

[Figure S6. The overall response rate (ORR) on day 28 and at any time of vedolizumab versus best available treatments (BATs) in total patients (A); the ORR on day 28 and at any time of vedolizumab versus BATs in patients with stage 3 to 4 gastrointestinal graft-versus-host disease (B). Overall response included complete response (blue) plus partial response (orange) rates.	26](#_Toc234506119)

[Figure S7. Comparison of ≥ 1 type of infection between vedolizumab and BATs group.	27](#_Toc234506120)

[Figure S8. 2-year probability of clinical outcomes after vedolizumab and best available treatments (BATs) (A) OS, (B) NRM, (C) DFS, and (D) relapse.	28](#_Toc234506121)

[Figure S9. 2-year probability of clinical outcomes after vedolizumab and best available treatments (BATs) in the inverse probability of treatment weighting analysis: (A) OS, (B) NRM, (C) DFS, and (D) relapse.	29](#_Toc234506122)

[Figure S10. 2-year probability of clinical outcomes after vedolizumab and best available treatments (BATs) in propensity score matching analysis: (A) OS, (B) NRM, (C) DFS, and (D) relapse.	30](#_Toc234506123)

# Supplemental Methods

**Response assessments for aGVHD treatments**

Complete response (CR) is defined as a score of 0 for the aGVHD grading in all evaluable organs that indicates complete resolution of all signs and symptoms of aGVHD in all evaluable organs without administration of additional systemic therapy for any earlier progression, mixed response or non-response of aGVHD. Partial response (PR) is defined as improvement of 1 stage in 1 or more organs involved with aGVHD signs or symptoms without progression in other organs or sites without administration of additional systemic therapy for an earlier progression, mixed response or non-response of aGVHD.

Overall response rate (ORR) includes CR plus PR rates. Lack of response is defined as no response, mixed response, or progression. No response is defined as absence of improvement in any organ involved by aGVHD, without worsening in any involved organ. Progression is defined as worsening in 1 or more organs by 1 or more stages without improvement in any involved organ.

Particularly, patients requiring additional systemic therapy for aGVHD will be classified as lack of response.

**Data collection**

Required information was obtained through institutional electronic medical records, including demographics, underlying diagnosis, transplant regimen (e.g. donor type, conditioning regimen and GVHD prophylaxis), aGVHD organ involvement and severity^1^, details of vedolizumab administration (e.g., dose, treatment response and toxicity), and survival outcomes.

**Endpoints and definition**

Overall survival (OS): time from vedolizumab treatment to death from any cause;

Disease-free survival (DFS): survival period with continuous complete remission after vedolizumab treatment;

Non-relapse mortality (NRM): time from vedolizumab treatment to death from any cause without recurrence of underlying disease;

Relapse: time from vedolizumab treatment to relapse

Reference

1.	MacMillan ML, Robin M, Harris AC, et al. A refined risk score for acute graft-versus-host disease that predicts response to initial therapy, survival, and transplant-related mortality. *Biol Blood Marrow Transplant*. Apr 2015;21(4):761-7.

# Supplemental Tables

## Table S1. Characteristics of GVHD of patients receiving vedolizumab.

| **Characteristics of aGVHD** | ***n* = 100** |
| --- | --- |
| Median time from transplantation to diagnosis of aGVHD, *day* (range) | 28 (11−97) |
| Median time from diagnosis of SR-aGVHD to vedolizumab treatment, *day* (range) | 13 (4−121) |
| Severity of skin aGVHD at diagnosis, *n* (%) |  |
| Stage 0 | 27 (27.0) |
| Stage 1 | 33 (33.0) |
| Stage 2 | 25 (25.0) |
| Stage 3 | 10 (10.0) |
| Stage 4 | 5 (5.0) |
| Severity of upper gastrointestinal aGVHD at diagnosis, *n* (%) |  |
| Stage 0 | 78 (78.0) |
| Stage 1 | 22 (22.0) |
| Severity of lower gastrointestinal aGVHD at diagnosis, *n* (%) |  |
| Stage 0 | 9 (9.0) |
| Stage 1 | 25 (25.0) |
| Stage 2 | 27 (27.0) |
| Stage 3 | 19 (19.0) |
| Stage 4 | 20 (20.0) |
| Severity of liver aGVHD at diagnosis, *n* (%) |  |
| Stage 0 | 63 (63.0) |
| Stage 1 | 7 (7.0) |
| Stage 2 | 14 (14.0) |
| Stage 3 | 8 (8.0) |
| Stage 4 | 8 (8.0) |
| Overall severity of aGVHD at diagnosis, *n* (%) |  |
| Grade I | 6 (6.0) |
| Grade II | 27 (27.0) |
| Grade III | 41 (41.0) |
| Grade IV | 26 (26.0) |
| Refined Minnesota aGVHD risk score at aGVHD diagnosis, *n* (%) |  |
| Standard risk | 34 (34.0) |
| High risk | 66 (66.0) |
| Severity of skin aGVHD before vedolizumab treatment, *n* (%) |  |
| Stage 0 | 49 (49.0) |
| Stage 1 | 26 (26.0) |
| Stage 2 | 16 (16.0) |
| Stage 3 | 6 (6.0) |
| Stage 4 | 3 (3.0) |
| Severity of upper gastrointestinal aGVHD before vedolizumab treatment, *n* (%) |  |
| Stage 0 | 73 (73.0) |
| Stage 1 | 27 (27.0) |
| Severity of lower gastrointestinal aGVHD before vedolizumab treatment, *n* (%) |  |
| Stage 0 | 0 |
| Stage 1 | 14 (14.0) |
| Stage 2 | 26 (26.0) |
| Stage 3 | 25 (25.0) |
| Stage 4 | 35 (35.0) |
| Severity of liver aGVHD before vedolizumab treatment, *n* (%) |  |
| Stage 0 | 71 (71.0) |
| Stage 1 | 6 (6.0) |
| Stage 2 | 11 (11.0) |
| Stage 3 | 8 (8.0) |
| Stage 4 | 4 (4.0) |
| Overall severity of aGVHD before vedolizumab treatment, *n* (%) |  |
| Grade II | 14 (14.0) |
| Grade III | 50 (50.0) |
| Grade IV | 36 (36.0) |
| Refined Minnesota aGVHD risk score before vedolizumab treatment, *n* (%) |  |
| Standard risk | 16 (16.0) |
| High risk | 84 (84.0) |

Abbreviation: aGVHD, acute graft-versus-host disease; SR, steroid-refractory.

## Table S2. The association between time interval from SR-aGVHD diagnosis to vedolizumab treatment and ORR

|  | Time interval from SR-aGVHD diagnosis to vedolizumab treatment | | |
| --- | --- | --- | --- |
|  | ≤ 5 days | > 5 and ≤ 7 days | *P* |
| Stage 3-4 SR-GI-aGVHD, *n* | 3 | 20 |  |
| ORR on day 28, *n* (%) | 2 (66.7%) | 19 (95.0%) | 0.249 |
| ORR at any time, *n* (%) | 3 (100%) | 19 (95.0%) | 1.000 |
| Stage 2 SR-GI-aGVHD, *n* | 0 | 3 |  |
| ORR on day28, *n* (%) | NA | 3 (100%) | NA |
| ORR at any time, *n* (%) | NA | 3 (100%) | NA |

Abbreviation: SR-GI-aGVHD, steroid-refractory gastrointestinal acute graft-versus-host disease; ORR, overall response rate; NA, not applicable.

## Table S3. Patient characteristics between those receiving vedolizumab treatment ≤ 7 days and > 7 days after SR-aGVHD diagnosis

|  | Time interval from SR-aGVHD diagnosis to vedolizumab treatment | | |
| --- | --- | --- | --- |
| **Variable** | ≤ 7 days  (*n* = 26) | > 7 days  (*n* = 74) | *P* |
| Median age, *year* (range) | 36 (6-66) | 32 (2-62) | 0.262 |
| Female sex, *n* (%) | 12 (46.15) | 38 (51.35) | 0.648 |
| Underlying disease, *n* (%) |  |  | 0.503 |
| Hematologic malignancies | 25 (96.15) | 66 (89.19) |  |
| Nonmalignant hematologic disease | 1 (3.85) | 8 (10.81) |  |
| HCT-CI score, *n* (%) |  |  | 0.729 |
| Low risk | 21 (80.77) | 56 (75.68) |  |
| Intermediate risk | 4 (15.38) | 16 (21.62) |  |
| High risk | 1 (3.85) | 2 (2.70) |  |
| Donor–recipient relationship, *n* (%) |  |  | 0.405 |
| Matched sibling donor | 6 (23.08) | 10 (13.51) |  |
| Alternative donor | 20 (76.92) | 64 (86.49) |  |
| Donor-recipient sex matched, *n* (%) |  |  | 0.149 |
| Others | 21 (80.77) | 69 (93.24) |  |
| Female to male | 5 (19.23) | 5 (6.76) |  |
| Conditioning regimen, *n* (%) |  |  | 0.232 |
| Chemotherapy-based regimen | 22 (84.62) | 54 (72.97) |  |
| Total-body-irradiation-based regimen | 4 (15.38) | 20 (27.03) |  |
| Steroid refractory type, *n* (%) |  |  | 0.664 |
| Steroid resistant | 24 (92.31) | 64 (86.49) |  |
| Steroid dependent | 2 (7.69) | 10 (13.51) |  |
| Severity of GI-aGVHD before treatment, *n* (%) |  |  | 0.726 |
| Stage 2 | 3 (11.54) | 11 (14.86) |  |
| Stage 3 | 15 (57.69) | 36 (48.65) |  |
| Stage 4 | 8 (30.77) | 27 (36.49) |  |

Abbreviation: BATs, best available treatments; IPTW, inverse probability of treatment weighting; PSM, propensity score matching; GI-aGVHD, gastrointestinal acute graft-versus-host disease; HCT-CI, hematopoietic cell transplantation-comorbidity index.

## Table S4. The overall response on day 28 and at any time of different subgroups in patients with gastrointestinal (GI) involvement.

|  | Response on day 28 | | | P |  | Response at any time (%) | | | P |
| --- | --- | --- | --- | --- | --- | --- | --- | --- | --- |
|  | CRR | PRR | ORR (95% CI) |  |  | CRR | PRR | ORR (95% CI) |  |
| **Stage 2 GI** |  |  |  | 0.439 |  |  |  |  | NS. |
| Without prior second-line treatment (n=5) | 100% | 0 | 100% |  |  | 100% | 0 | 100% |  |
| With prior second-line treatment (n=9) | 66.7% | 22.2% | 88.9% (68.4-100.0%) |  |  | 77.8% | 22.2% | 100% |  |
| **Stage 3 GI** |  |  |  | 0.775 |  |  |  |  | 0.369 |
| Without prior second-line treatment (n=4) | 75.0% | 0 | 75.0% (32.6-100.0%) |  |  | 100% | 0 | 100% |  |
| With prior second-line treatment (n=47) | 61.7% | 6.4% | 68.1% (54.8-81.4%) |  |  | 66.0% | 17.0% | 83.0% (72.3-93.7%) |  |
| **Stage 4 GI** |  |  |  |  |  |  |  |  |  |
| With prior second-line treatment (n=35) | 37.1% | 20.0% | 57.1% (40.7-73.5%) |  |  | 66.0% | 17.0% | 83.0% (70.6-95.4%) |  |
| **Stage 2 GI** |  |  |  | 0.299 |  |  |  |  | NS. |
| Monotherapy (n=7) | 100% | 0 | 100% |  |  | 100% | 0 | 100% |  |
| Combined therapy (n=7) | 57.1% | 28.6% | 85.7% (59.8-100.0%) |  |  | 71.4% | 28.6% | 100% |  |
| **Stage 3 GI** |  |  |  | 0.889 |  |  |  |  | 0.154 |
| Monotherapy (n=9) | 66.7% | 0 | 66.7% (35.9-97.5%) |  |  | 77.8% | 22.2% | 100% |  |
| Combined therapy (n=42) | 61.9% | 7.1% | 69.0% (55.0-83.0%) |  |  | 66.7% | 14.3% | 81.0% (69.1-92.9%) |  |
| **Stage 4 GI** |  |  |  | 0.393 |  |  |  |  | 0.466 |
| Monotherapy (n=7) | 28.6% | 14.3% | 42.9% (6.2-79.6%) |  |  | 28.6% | 28.6% | 57.1% (20.4-93.8%) |  |
| Combined therapy (n=28) | 39.3% | 21.4% | 60.7% (42.6-78.8%) |  |  | 50.0% | 21.4% | 71.4% (54.7-88.1%) |  |

Abbreviation: CRR, complete response rate; PRR, partial response rate; ORR, overall response rate; ORR was defined as CR plus PR.

## Table S5. Patient characteristics between monotherapy and combined therapy groups.

| **Variable** | **Monotherapy (*n* = 23)** | **Combined therapy (*n* = 77)** | ***P*** |
| --- | --- | --- | --- |
| Median age at HSCT, *year* (range) | 24 (3−59) | 31 (2−66) | 0.169 |
| Female sex, *n* (%) | 16 (69.6) | 34 (44.2) | 0.032 |
| Underlying disease, *n* (%) |  |  | 0.440 |
| Hematologic malignancies | 20 (87.0) | 71 (92.2) |  |
| Nonmalignant hematologic disease | 3 (13.0) | 6 (7.8) |  |
| HCT-CI score, *n* (%) |  |  | 0.309 |
| Low risk | 15 (65.2) | 62 (80.5) |  |
| Intermediate risk | 7 (30.4) | 13 (16.9) |  |
| High risk | 1 (4.3) | 2 (2.6) |  |
| Donor–recipient relationship, *n* (%) |  |  | 0.923 |
| Mother–child | 1 (4.3) | 3 (3.9) |  |
| Others | 22 (95.7) | 74 (96.1) |  |
| Donor-recipient sex matched, *n* (%) |  |  | 0.812 |
| Female to male | 2 (8.7) | 8 (10.4) |  |
| Others | 21 (91.3) | 69 (89.6) |  |
| Donor type, *n* (%) |  |  | 0.276 |
| Matched sibling donor | 2 (8.7) | 14 (18.2) |  |
| Alternative donor | 21 (91.3) | 63 (81.8) |  |
| Conditioning regimen, *n* (%) |  |  | 0.013 |
| Chemotherapy based regimen | 13 (56.5) | 63 (81.8) |  |
| Total-body-irradiation-based regimen | 10 (43.5) | 14 (18.2) |  |
| Median time from diagnosis of aGVHD to vedolizumab treatment, day (range) | 32 (6-120) | 11 (4-121) | 0.003 |
| Overall severity of gastrointestinal aGVHD before vedolizumab treatment, *n* (%) |  |  | 0.010 |
| Stage 2 | 7 (30.4) | 7 (9.1) |  |
| Stage 3-4 | 16 (69.6) | 70 (90.9) |  |
| Steroid refractory type, *n* (%) |  |  | 0.578 |
| Steroid resistant | 21 (91.3) | 67 (87.0) |  |
| Steroid dependent | 2 (8.7) | 10 (13.0) |  |

Abbreviation:HSCT, hematopoietic stem cell transplantation; HCT-CI, hematopoietic cell transplantation-comorbidity index, aGVHD, acute graft-versus-host disease.

## Table S6. Univariate analysis for response of GI-aGVHD and clinical outcomes after vedolizumab treatment.

| **Outcome** | **HR (95% CI)** | ***P*** |
| --- | --- | --- |
| **Lack of response at 28 day** |  |  |
| Underlying disease |  |  |
| Hematologic malignancies | 1.00 |  |
| Nonmalignant hematologic disease | 1.80 (0.45–7.21) | 0.407 |
| Donor–recipient relationship |  |  |
| Identical sibling donor | 1.00 |  |
| Alternative donor | 0.75 (0.25–2.27) | 0.608 |
| HCT-CI score |  |  |
| 0 (low risk) | 1.00 |  |
| ≥ 1 (intermediate/high risk) | 1.51 (0.57–3.98) | 0.405 |
| Conditioning regimen |  |  |
| Chemotherapy-based regimen | 1.00 |  |
| Total-body-irradiation-based regimen | 0.64 (0.23–1.81) | 0.401 |
| Severity of GI-aGVHD before vedolizumab treatment |  |  |
| Stage 2 | 1.00 |  |
| Stage 3 to 4 | 7.33 (0.91–58.71) | 0.061 |
| Steroid refractory type |  |  |
| Steroid resistant | 1.00 |  |
| Steroid dependent | 2.38 (0.70–8.08) | 0.163 |
| Other second-line treatment before vedolizumab |  |  |
| No | 1.00 |  |
| Yes | 4.13 (0.49–34.56) | 0.190 |
| Type of vedolizumab treatment |  |  |
| Monotherapy | 1.00 |  |
| Combined therapy | 1.10 (0.40–3.01) | 0.855 |
| Time to vedolizumab after aGVHD |  |  |
| >7 days | 1.00 |  |
| ≤ 7 days | 8.18 (1.80–37.23) | 0.007 |
| Liver involvement at aGVHD diagnosis |  |  |
| No | 1.00 |  |
| Yes | 1.03 (0.43–2.46) | 0.943 |
| Transplant centers |  |  |
| Enrolled ≥ median | 1.00 |  |
| Enrolled < median | 0.74 (0.30–1.82) | 0.513 |
| GVHD prophylaxis |  |  |
| CNI + MMF + MTX | 1.00 |  |
| CNI + MMF + MTX + ATG | 1.16 (0.44–3.05) | 0.760 |
| CNI + MMF + MTX + ATG + PTCY | 1.19 (0.09–15.04) | 0.894 |
| **Lack of response at any time** |  |  |
| Underlying disease |  |  |
| Hematologic malignancies | 1.00 |  |
| Nonmalignant hematologic disease | 0.51 (0.06–4.32) | 0.534 |
| Donor–recipient relationship |  |  |
| Identical sibling donor | 1.00 |  |
| Alternative donor | 1.78 (0.37–8.57) | 0.475 |
| HCT-CI score |  |  |
| 0 (low risk) | 1.00 |  |
| ≥ 1 (intermediate/high risk) | 1.25 (0.40–3.94) | 0.703 |
| Conditioning regimen |  |  |
| Chemotherapy-based regimen | 1.00 |  |
| Total-body-irradiation-based regimen | 0.32 (0.07–1.48) | 0.143 |
| Severity of GI-aGVHD before vedolizumab treatment |  |  |
| Stage 2 | NA |  |
| Stage 3 to 4 | NA | NA |
| Steroid refractory type |  |  |
| Steroid resistant | 1.00 |  |
| Steroid dependent | 2.43 (0.65–9.13) | 0.188 |
| Other second-line treatment before vedolizumab |  |  |
| No | NA |  |
| Yes | NA | NA |
| Type of vedolizumab treatment |  |  |
| Monotherapy | 1.00 |  |
| Combined therapy | 1.75 (0.46–6.63) | 0.411 |
| Time to vedolizumab after aGVHD |  |  |
| >7 days | 1.00 |  |
| ≤7 days | 8.04 (1.02–63.52) | 0.048 |
| Liver involvement at aGVHD diagnosis |  |  |
| No | 1.00 |  |
| Yes | 1.70 (0.62–4.68) | 0.301 |
| Transplant centers |  |  |
| Enrolled ≥ median | 1.00 |  |
| Enrolled < median | 0.91 (0.31–2.68) | 0.867 |
| GVHD prophylaxis |  |  |
| CNI + MMF + MTX | 1.00 |  |
| CNI + MMF + MTX + ATG | 1.57 (0.47–5.24) | 0.464 |
| CNI + MMF + MTX + ATG + PTCY | NA | NA |
| **Infection (≥ 1 events)** |  |  |
| Underlying disease |  |  |
| Hematologic malignancies | 1.00 |  |
| Nonmalignant hematologic disease | 0.42 (0.05–3.52) | 0.422 |
| Donor–recipient relationship |  |  |
| Identical sibling donor | 1.00 |  |
| Alternative donor | 0.39 (0.12–1.24) | 0.110 |
| HCT-CI score |  |  |
| 0 (low risk) | 1.00 |  |
| ≥ 1 (intermediate/high risk) | 0.27 (0.06–1.26) | 0.096 |
| Conditioning regimen |  |  |
| Chemotherapy-based regimen | 1.00 |  |
| Total-body-irradiation-based regimen | 0.25 (0.05–1.18) | 0.081 |
| Severity of GI-aGVHD before vedolizumab treatment |  |  |
| Stage 2 | 1.00 |  |
| Stage 3 to 4 | NA | NA |
| Steroid refractory type |  |  |
| Steroid resistant | 1.00 |  |
| Steroid dependent | 2.98 (0.84–10.56) | 0.090 |
| Other second-line treatment before vedolizumab |  |  |
| No | NA |  |
| Yes | NA | NA |
| Type of vedolizumab treatment |  |  |
| Monotherapy | 1.00 |  |
| Combined therapy | 8.25 (1.05–65.09) | 0.045 |
| Time to vedolizumab after aGVHD |  |  |
| >7 days | 1.00 |  |
| ≤7 days | 0.24 (0.09–0.65) | 0.005 |
| Liver involvement at aGVHD diagnosis |  |  |
| No | 1.00 |  |
| Yes | 5.60 (2.03–15.40) | <.001 |
| Transplant centers |  |  |
| Enrolled ≥ median | 1.00 |  |
| Enrolled < median | 1.19 (0.41–3.40) | 0.752 |
| GVHD prophylaxis |  |  |
| CNI + MMF + MTX | 1.00 |  |
| CNI + MMF + MTX + ATG | 0.41 (0.15–1.14) | 0.088 |
| CNI + MMF + MTX + ATG + PTCY | 1.00 (0.08–12.56) | 1.000 |
| **Treatment failure as defined by OS** |  |  |
| Underlying disease |  |  |
| Hematologic malignancies | 1.00 |  |
| Nonmalignant hematologic disease | 0.88 (0.27–2.87) | 0.827 |
| Donor–recipient relationship |  |  |
| Identical sibling donor | 1.00 |  |
| Alternative donor | 1.07 (0.44–2.58) | 0.889 |
| HCT-CI score |  |  |
| 0 (low risk) | 1.00 |  |
| ≥1 (intermediate/high risk) | 0.51 (0.20–1.33) | 0.172 |
| Conditioning regimen |  |  |
| Chemotherapy-based regimen | 1.00 |  |
| Total-body-irradiation-based regimen | 0.88 (0.38–2.03) | 0.758 |
| Severity of GI-aGVHD before vedolizumab treatment |  |  |
| Stage 2 | 1.00 |  |
| Stage 3 to 4 | 4.59 (0.63–33.59) | 0.134 |
| Steroid refractory type |  |  |
| Steroid resistant | 1.00 |  |
| Steroid dependent | 2.09 (0.86–5.09) | 0.104 |
| Other second-line treatment before vedolizumab |  |  |
| No | NA |  |
| Yes | NA | NA |
| Type of vedolizumab treatment |  |  |
| Monotherapy | 1.00 |  |
| Combined therapy | 1.82 (0.70–4.71) | 0.218 |
| Time to vedolizumab after aGVHD |  |  |
| >7 days | 1.00 |  |
| ≤7 days | 1.99 (0.82–4.83) | 0.128 |
| Liver involvement at aGVHD diagnosis |  |  |
| No | 1.00 |  |
| Yes | 4.44 (2.22–8.89) | <.001 |
| Transplant centers |  |  |
| Enrolled ≥ median | 1.00 |  |
| Enrolled < median | 0.91 (0.43–1.92) | 0.807 |
| GVHD prophylaxis |  |  |
| CNI + MMF + MTX | 1.00 |  |
| CNI + MMF + MTX + ATG | 1.18 (0.55–2.54) | 0.675 |
| CNI + MMF + MTX + ATG + PTCY | NA | NA |
| **Treatment failure as defined by DFS** |  |  |
| Underlying disease |  |  |
| Hematologic malignancies | 1.00 |  |
| Nonmalignant hematologic disease | 0.77 (0.24–2.54) | 0.672 |
| Donor–recipient relationship |  |  |
| Identical sibling donor | 1.00 |  |
| Alternative donor | 1.08 (0.45–2.61) | 0.862 |
| HCT-CI score |  |  |
| 0 (low risk) | 1.00 |  |
| ≥1 (intermediate/high risk) | 0.66 (0.29–1.53) | 0.333 |
| Conditioning regimen |  |  |
| Chemotherapy-based regimen | 1.00 |  |
| Total-body-irradiation-based regimen | 0.81 (0.35–1.86) | 0.625 |
| Severity of GI-aGVHD before vedolizumab treatment |  |  |
| Stage 2 | 1.00 |  |
| Stage 3 to 4 | 4.89 (0.67–35.76) | 0.118 |
| Steroid refractory type |  |  |
| Steroid resistant | 1.00 |  |
| Steroid dependent | 2.01 (0.83–4.87) | 0.124 |
| Other second-line treatment before vedolizumab |  |  |
| No | NA |  |
| Yes | NA | NA |
| Type of vedolizumab treatment |  |  |
| Monotherapy | 1.00 |  |
| Combined therapy | 2.02 (0.78–5.20) | 0.147 |
| Time to vedolizumab after aGVHD |  |  |
| >7 days | 1.00 |  |
| ≤7 days | 1.77 (0.77–4.07) | 0.176 |
| Liver involvement at aGVHD diagnosis |  |  |
| No | 1.00 |  |
| Yes | 4.18 (2.11–8.31) | <.001 |
| Transplant centers |  |  |
| Enrolled ≥ median | 1.00 |  |
| Enrolled < median | 0.88 (0.43–1.80) | 0.725 |
| GVHD prophylaxis |  |  |
| CNI + MMF + MTX | 1.00 |  |
| CNI + MMF + MTX + ATG | 1.12 (0.54–2.33) | 0.765 |
| CNI + MMF + MTX + ATG + PTCY | NA | NA |
| **Non-relapse mortality** |  |  |
| Underlying disease |  |  |
| Hematologic malignancies | 1.00 |  |
| Nonmalignant hematologic disease | 1.15 (0.35–3.84) | 0.817 |
| Donor–recipient relationship |  |  |
| Identical sibling donor | 1.00 |  |
| Alternative donor | 0.96 (0.36–2.54) | 0.931 |
| HCT-CI score |  |  |
| 0 (low risk) | 1.00 |  |
| ≥ 1 (intermediate/high risk) | 0.37 (0.11–1.25) | 0.110 |
| Conditioning regimen |  |  |
| Chemotherapy-based regimen | 1.00 |  |
| Total-body-irradiation-based regimen | 0.58 (0.20–1.70) | 0.324 |
| Severity of GI-aGVHD before vedolizumab treatment |  |  |
| Stage 2 | NA |  |
| Stage 3 to 4 | NA | NA |
| Steroid refractory type |  |  |
| Steroid resistant | 1.00 |  |
| Steroid dependent | 2.21 (0.83–5.91) | 0.114 |
| Other second-line treatment before vedolizumab |  |  |
| No | NA |  |
| Yes | NA | NA |
| Type of vedolizumab treatment |  |  |
| Monotherapy | 1.00 |  |
| Combined therapy | 1.38 (0.52–3.66) | 0.519 |
| Time to vedolizumab after aGVHD |  |  |
| >7 days | 1.00 |  |
| ≤7 days | 2.44 (0.84–7.09) | 0.102 |
| Liver involvement at aGVHD diagnosis |  |  |
| No | 1.00 |  |
| Yes | 5.66 (2.55–12.55) | <0.001 |
| Transplant centers |  |  |
| Enrolled ≥ median | 1.00 |  |
| Enrolled < median | 0.90 (0.39–2.08) | 0.809 |
| GVHD prophylaxis |  |  |
| CNI + MMF + MTX | 1.00 |  |
| CNI + MMF + MTX + ATG | 0.99 (0.43–2.28) | 0.981 |
| CNI + MMF + MTX + ATG + PTCY | NA | NA |

Abbreviation: GI, gastrointestinal, GVHD, graft-versus-host disease; MTX, methotrexate; CNI, calcineurin inhibitor; MMF, mycophenolate mofetil; ATG, anti-thymocyte globulin; PTCY, post-transplant cyclophosphamide. ORR, overall response rate; OS, overall survival; NRM, non-relapse mortality; DFS disease-free survival; NA, not applicable.

## Table S7. Characteristics of cGVHD.

| **Characteristics of cGVHD** | ***n* = 22** |
| --- | --- |
| Median time from the beginning of vedolizumab treatment to cGVHD, day (range) | 77 (26-473) |
| Grade of cGVHD, *n* (%) |  |
| Mild | 7 (31.8) |
| Moderate | 9 (40.9) |
| Severe | 6 (27.3) |
| Site of cGVHD, *n* (%) |  |
| Skin | 15 (68.2) |
| Mouth | 10 (45.5) |
| Eye | 8 (36.4) |
| Gastrointestinal, *n* (%) | 11 (55.0) |
| Liver | 10 (45.5) |
| Lung | 5 (23.8) |
| Others | 3 (13.6) |
| Number of sites, *n* (%) |  |
| 1 | 4 (18.2) |
| 2 | 6 (27.3) |
| ≥ 3 | 12 (54.5) |

Abbreviation: cGVHD, chronic graft-versus-host disease.

## Table S8. New onset infections after vedolizumab treatment in subgroup analysis.

| **Infection categories** | **Line of vedolizumab treatment** | |  | **Type of vedolizumab treatment** | |  | **Severity of aGVHD** | |  |
| --- | --- | --- | --- | --- | --- | --- | --- | --- | --- |
|  | **Second line (*n*=9)** | **≥ second line (*n*=91)** | ***P*** | **Monotherapy (*n*=23)** | **Combined therapy (*n*=77)** | ***P*** | **Stage 2 GI-aGVHD (*n*=14)** | **Stage 3 – 4 GI-aGVHD (*n*=86)** | ***P*** |
| Viral infection, *n* (%) | 0 | 19 (20.9%) | 0.128 | 2 (8.7%) | 17 (22.1%) | 0.151 | 0 | 19 (22.1%) | 0.051 |
| Bacterial infection, *n* (%) | 0 | 4 (4.4%) | 0.521 | 1 (4.3%) | 3 (3.9%) | 0.923 | 0 | 4 (4.7%) | 0.410 |
| Fungal infection, *n* (%) | 0 | 4 (4.4%) | 0.521 | 0 | 4 (5.2%) | 0.265 | 0 | 4 (4.7%) | 0.410 |
| Any infection (≥ 1 type), *n* (%) | 0 | 22 (24.2%) | 0.095 | 1 (4.3%) | 21 (27.3%) | 0.020 | 0 | 22 (25.6%) | 0.032 |
| Multiple infection (≥ 2 types), *n* (%) | 0 | 3 (3.3%) | 1.000 | 0 | 3 (3.9%) | 1.000 | 0 | 3 (3.5%) | 1.000 |

Abbreviation: GI, gastrointestinal; aGVHD acute graft-versus-host disease.

## Table S9. Causes of death.

| **Causes of death** | ***n* (%)** |
| --- | --- |
| Relapse | 7 (7.0) |
| Non-relapse mortality |  |
| Infection | 14 (14.0) |
| Graft-versus-host disease | 5 (5.0) |
| Thrombotic microangiopathy | 4 (4.0) |
| Gastrointestinal hemorrhage | 1 (1.0) |
| Heart failure | 1 (1.0) |
| Intracerebral hemorrhage | 1 (1.0) |

## Table S10. Comparison of overall response rate between vedolizumab and each individual intervention in the BATs group.

|  |  | **Lack of overall response on day 28** | | **Lack of overall response at any time** | |
| --- | --- | --- | --- | --- | --- |
|  | No. of patients, *n* (%) | HR (95%CI) | *P* | HR (95%CI) | *P* |
| **Vedolizumab** | 100 (100) | 1.00 |  | 1.00 |  |
| **BATs monotherapy** | 341 (83.0) |  |  |  |  |
| Basiliximab | 246 (59.9) | 1.16 (0.71–1.91) | 0.550 | 1.56 (0.88–2.77) | 0.127 |
| Mycophenolate mofetil | 25 (6.1) | 2.30 (0.95–5.61) | 0.066 | 2.84 (1.11–7.30) | 0.030 |
| Etanercept | 18 (4.4) | 1.06 (0.37–3.09) | 0.911 | 1.22 (0.36–4.12) | 0.751 |
| Ruxolitinib | 21 (5.1) | 1.59 (0.61–4.17) | 0.342 | 2.62 (0.95–7.22) | 0.062 |
| Mesenchymal stromal cells | 31 (7.5) | 1.17 (0.50–2.73) | 0.718 | 1.48 (0.58–3.82) | 0.415 |
| **Combination BATs** | 70 (17.0) |  |  |  |  |
| Two-drug regimens | 64 (15.6) | 1.65 (0.86–3.16) | 0.129 | 1.09 (0.49–2.39) | 0.836 |
| Three-drug regimens | 6 (1.5) | 1.06 (0.18–6.11) | 0.946 | 2.13 (0.36–12.51) | 0.402 |

Abbreviation: BATs, best available treatments; two-drug regimens included basiliximab plus sirolimus, basiliximab plus mycophenolate mofetil and basiliximab plus ruxolitinib; three-drug was basiliximab+ mycophenolate mofetil+ ruxolitinib.

# Supplemental Figures

## Figure S1. Swimmers plot showing duration of treatment response (complete response or partial response) and survival status in patients treated with vedolizumab. Each horizontal bar represents one patient; bar length indicates time from vedolizumab initiation to last follow-up or event.

## Figure S2. Overall response rate (ORR) of patients receiving other second-line treatments before vedolizumab on day 28 (A) and at any time (B). Overall response included complete response (blue) plus partial response (orange) rates.

## Figure S3. Overall response rate (ORR) of patients who received other second-line treatments combined with vedolizumab on day 28 (A) and at any time (B). ORR included complete response (blue) plus partial response (orange) rates

## Figure S4. Overall response rate (ORR) of skin and liver after vedolizumab on day 28 (A) and at any time (B). Overall response included complete response (blue) plus partial response (orange) rates.

## Figure S5. Overall response rate (ORR) of patients with different severity of overall aGVHD after vedolizumab on day 28 and at any time. ORR included complete response (blue) plus partial response (orange) rates.

## Figure S6. The overall response rate (ORR) on day 28 and at any time of vedolizumab versus best available treatments (BATs) in total patients (A); the ORR on day 28 and at any time of vedolizumab versus BATs in patients with stage 3 to 4 gastrointestinal graft-versus-host disease (B). Overall response included complete response (blue) plus partial response (orange) rates.

## Figure S7. Comparison of ≥ 1 type of infection between vedolizumab and BATs group.

BATs, best available treatments; IPTW, inverse probability of treatment weighting; PSM, propensity score matching.

## Figure S8. 2-year probability of clinical outcomes after vedolizumab and best available treatments (BATs) (A) OS, (B) NRM, (C) DFS, and (D) relapse.

OS, overall survival; NRM, non-relapse mortality; DFS disease-free survival.

## Figure S9. 2-year probability of clinical outcomes after vedolizumab and best available treatments (BATs) in the inverse probability of treatment weighting analysis: (A) OS, (B) NRM, (C) DFS, and (D) relapse.

OS, overall survival; NRM, non-relapse mortality; DFS disease-free survival.

## Figure S10. 2-year probability of clinical outcomes after vedolizumab and best available treatments (BATs) in propensity score matching analysis: (A) OS, (B) NRM, (C) DFS, and (D) relapse.

OS, overall survival; NRM, non-relapse mortality; DFS disease-free survival.
